# The global antimicrobial resistance response effort must not exclude marginalised populations

**DOI:** 10.1186/s41182-023-00524-w

**Published:** 2023-06-07

**Authors:** Yusuff Adebayo Adebisi, Isaac Olushola Ogunkola

**Affiliations:** 1 Nuffield Department of Population Health, University of Oxford, Oxford, UK; 2 Global Health Focus, Kigali, Rwanda; 3 Department of Public Health, University of Calabar, Calabar, Cross River Nigeria

## Abstract

Antimicrobial resistance (AMR), a rising global health crisis causing about 700,000 deaths annually and potentially 10 million deaths by 2050, disproportionately impacts marginalised populations. Due to socioeconomic, ethnic, geographic, and other barriers, these communities often have restricted healthcare access, compounding the AMR threat. Unequal access to effective antibiotics, inadequate living conditions, and a lack of awareness exacerbate the crisis in marginalised communities, making them more susceptible to AMR. A broader, inclusive response is needed to ensure equitable access to antibiotics, improved living conditions, education, and policy changes to challenge the root socio-economic disparities. Ignoring marginalised populations in the fight against AMR is both a moral and strategic failure. Therefore, inclusivity must be a central tenet in combating AMR. This article not only critically dissects this prevailing oversight but also urgently calls for comprehensive action to address this significant shortcoming in our response efforts.

## To the Editor,

In the realm of public health, there is an inherent interplay between the global community and the individual, the majority, and the minority. This interplay is most visible when we are faced with a crisis that is both global and personal in its impact. The current situation of increasing antimicrobial resistance (AMR) is a case in point, causing about 700,000 deaths annually, and projections suggest that by 2050, AMR could lead to 10 million deaths per year, surpassing the current 8.2 million annual deaths from cancer [1, 2]. As the world grapples with this looming health crisis, one thing is clear: the global AMR effort should not, under any circumstances, exclude marginalised populations. This statement might seem self-evident, almost tautological. Yet, we find numerous instances, where this is not the case. This commentary offers an analysis of this oversight, and issues an emphatic call to action to address this glaring gap in our response efforts.

Marginalised populations, in this context, are those who experience systemic exclusion, often resulting in reduced access to healthcare due to factors such as socioeconomic status, ethnicity, geographic location, immigration status, and disability. These groups can include rural and remote communities, urban slums, ethnic and racial minorities, indigenous communities, and immigrant or refugee populations, each facing unique and compounded barriers that exacerbate the challenges associated with AMR [3]. This disparity becomes strikingly apparent when we examine the response to the escalating AMR threat. The rise of antibiotic-resistant bacteria is a worldwide health crisis, threatening to plunge us back into a pre-antibiotic era. The reasons for this crisis are manifold, from overuse and misuse of antibiotics in humans and animals to a lack of new drugs in development due to market failures [4, 5]. However, the impact is not distributed equally with the marginalised population likely to be disproportionately affected. The underprivileged are often left dealing with outdated or ineffective drugs, or worse, counterfeit ones, further accelerating AMR [7]. Moreover, essential antibiotics, while generally inexpensive to produce, can either be difficult to access for impoverished communities or easily available over the counter in some settings, leading to misuse and fuelling resistance [6, 7]. For instance, the fourth report of the Global Evidence Review on Health and Migration has shown that given the variability in health systems across countries with different income levels, refugees and migrants face a wide range of barriers in obtaining health services and, by extension, in accessing and using antibiotics [8].

Furthermore, we must also recognize the role of social determinants of health [6]. Overcrowding, poor sanitation, lack of clean water—all these conditions prevalent in marginalised communities create an environment conducive to the spread of infectious diseases and, hence, an increased need for antibiotics [9]. Researchers have previously highlighted how settings housing marginalised populations, such as prisons and other enclosed environments could serve as ecological reservoirs for antimicrobial-resistant strains if we fail to extend response efforts to these institutions [10]. In addition, the dearth of awareness and education about AMR in marginalised communities, coupled with language and cultural barriers, could exacerbate the problem. Insufficient awareness often leads to behaviours that amplify antibiotic resistance, such as non-adherence to prescribed treatment regimens, misuse of antibiotics for viral infections, sharing or selling of prescribed antibiotics, and self-medication due to lack of access to professional healthcare. Without proper knowledge, it is almost impossible to practice effective use of antibiotics, leading to inappropriate usage and the spread of resistant strains. To ensure the inclusion of the One Health approach in advancing AMR response efforts among marginalised populations, it is crucial to prioritize targeted education and community engagement, while also addressing healthcare disparities and fostering multi-sectoral collaborations that emphasize equity and access to healthcare services for both humans and animals.

Indigenous communities globally are more likely to live in poverty and have less access to quality healthcare [11]. This, along with other socio-economic factors, makes these populations more vulnerable to drug-resistant infections [12]. A study among indigenous population of central Australia highlighted an incidence of superficial skin and soft tissue infections at a rate of 18.9 per 1000 people years among indigenous populations compared to 2.9 per 1000 in non-indigenous groups, suggesting high antibiotic resistance [13]. This is corroborated by 57% of samples showing community-associated methicillin-resistant *S. aureus* [13]. A systematic review has also shown a high prevalence of AMR in a variety of bacterial species among displaced populations, including refugees [14]. An extensive study examining 43,770 pathogens from 37,276 individuals found migrants to have higher susceptibility to Enterobacterales infections (OR 1.42, 95% CI: 1.23–1.63), with an escalated risk of methicillin-resistant S. aureus (MRSA) in family-reunited migrants and refugees [15]. Notably, the risk of ciprofloxacin-resistant Enterobacterales infections was heightened in these groups, with women migrants showing a markedly increased prevalence of these pathogens [15]. The process of displacement itself significantly contributes to the transmission of AMR, largely due to crowded living conditions and limited healthcare access. Another study also revealed that diarrheagenic *Escherichia coli* (DEC) was detected in 58% of the children residing in the slum area, while it was found in only 17% of the control group (children who attended a private school of the same city) (*P* = 0.001) [16]. Furthermore, the study identified that 65% of the DEC strains exhibited resistance to at least one antimicrobial drug [16]. In addition, a concerning 46% of the strains showed resistance to two or more antimicrobial drugs [16]. All these findings highlight the urgent need for enhanced AMR control measures and further research to understand and address this pressing public health issue in vulnerable populations.

The WHO's Global Action Plan on Antimicrobial Resistance [17], while offering a comprehensive approach to the AMR threat, lacks a specific focus on marginalised and vulnerable populations. The absence of explicit mention of these groups does not align with the WHO's overarching commitment to health equity and risks fostering a generalized approach in both global and national response plans. This omission inadvertently marginalises populations that already experience health inequities due to their unique circumstances and needs, and thus, might be more susceptible to AMR due to factors like inadequate access to quality healthcare, poor sanitation, and misuse of antimicrobials. In essence, the lack of targeted attention to marginalised groups in the Action Plan undermines the principles of health equity, because it does not fully address the specific challenges these populations face in dealing with AMR. Explicit inclusion of marginalised populations in the Action Plan is crucial as it can help cater to their specific needs and circumstances, thereby contributing to a more effective response to AMR. It can help tailor prevention strategies, surveillance, research, and educational campaigns that are more sensitive to the cultural, linguistic, socio-economic, and geographical specificities of these populations. Moreover, an inclusive approach aligns with the principles of health equity by ensuring that everyone, regardless of their socio-economic status, has a fair and just opportunity to curb, treat, and manage AMR. Hence, future revisions of the Action Plan must explicitly incorporate a focus on marginalised populations to ensure an effective and equitable fight against AMR.

The issue of AMR is not just a biological problem to be solved by scientists in labs. It is also a social problem that requires us to challenge our existing systems of health and economic inequality [6]. If the global response does not actively include marginalised populations, we risk leaving behind a significant portion of humanity in the battle against AMR, thereby undermining the whole effort. Implementing inclusive strategies in the global AMR response can be met with several challenges such as resource constraints in low-resource settings, cultural and language barriers in education, limited access to vaccinations and necessary antibiotics, and inadequate surveillance and reporting of AMR. In addition, there may be resistance towards implementing policy changes due to economic interests, and a lack of research focused on AMR in marginalised populations can hamper the development of tailored interventions.

To create an effective global response to AMR, we must ensure equitable access to antibiotics and healthcare in general. We need to engage with marginalised communities to provide education and awareness, building antibiotic stewardship from the ground up. Investments need to be made in improving living conditions and sanitation, reducing the need for antibiotic use in the first place. On a broader level, there is a need for policy changes that acknowledge and challenge the socio-economic disparities at the root of this problem. Policy changes can be implemented effectively through active collaboration between government bodies, healthcare organisations, and community leaders. Public health initiatives should be designed with cultural competence, incorporating local customs and language into educational materials to ensure understanding. For example, a "train-the-trainer" model can be employed, where community health workers are educated about AMR and antibiotic stewardship, who then pass on this knowledge to members of their communities, thereby ensuring a culturally sensitive and community-focused approach to public health education. Furthermore, mobile health clinics and telemedicine could also be utilised to reach remote and underserved communities. Addressing barriers to vaccination and promoting vaccination programs to prevent infections that typically necessitate antibiotic treatment can significantly mitigate the need for antibiotic usage, thereby reducing the risk of AMR. Alongside these efforts, it is imperative to encourage and prioritize research that focuses on AMR within marginalised populations, as this can provide critical insights into their unique challenges and inform the development of customized, effective interventions to tackle AMR in these communities.

If the goal is a healthier world where effective antibiotics are available to all, we must not forget that the 'all' includes the marginalised and the disenfranchised. Their exclusion from the global AMR response effort is not just a moral failing but also a strategic one, threatening to undo whatever gains we might achieve. Therefore, inclusivity must be at the heart of the fight against AMR.

## Data Availability

Not applicable.

## Abbreviations

AMR: Antimicrobial resistance
WHO: World Health Organization
